# Altered Effective Connectivity Patterns in MCI during Motion Detection Tasks

**DOI:** 10.1002/alz70861_109006

**Published:** 2025-12-23

**Authors:** Alina B Renli, Boxin Sun, Ming Gu, Voyko Kavcic, Tongtong Li, Bruno Giordani

**Affiliations:** ^1^ Michigan State University, East Lansing, MI USA; ^2^ Michigan Alzheimer's Disease Research Center, Ann Arbor, MI USA; ^3^ Wayne State University, Detroit, MI USA; ^4^ International Institute of Applied Gerontology, Ljubljana Slovenia; ^5^ University of Michigan, Ann Arbor, MI USA; ^6^ University of Michigan Medical School, Ann Arbor, MI USA

## Abstract

**Background:**

Existing work suggests that Alzheimer's pathology can affect the direction and intensity of information signaling in functional brain regions. The present study aims to explore the impact of cognitive impairment on effectivity connectivity, as well as identify possible discrepancies between people with normal cognition (NC) and mild cognitive impairment (MCI) patients.

**Method:**

Our research focuses on task‐based EEG (64‐channel), where participants were asked to perform a motion direction discrimination task. The current dataset includes 56 consensus‐diagnosed, community‐dwelling American seniors with subjective cognitive complaints (ages 60‐90 years, 28 NC and 28 MCI) recruited through the Wayne State University and Michigan Alzheimer’s Disease Research Center. For each participant, we evaluate the effective connectivity across all the possible EEG region pairs using causalized convergent cross‐mapping, a robust model for brain causality analysis based on state space reconstruction.

**Result:**

Our results show that during the motion detection task, MCI patients exhibit lower effective connectivity in some EEG‐region pairs, especially between left temporal (LT) and left central (LC), and right temporal (RT) and right central (RC). This may be attributed to the fact that the temporal lobe, which encompasses the hippocampus, is largely involved in the encoding and processing of memory and visual motion capture. Concurrently, MCI patients exhibit significantly higher effective connectivity in other region pairs.

**Conclusion:**

Altered effective connectivity measures may reflect compensatory brain activity among older individuals with MCI as they struggle to achieve comparable behavioral results to NC during the motion direction discrimination task. While effective connectivity may be decreased for MCI across certain region pairs, a significant increase in effective connectivity in some other regions pairs may be an indicator associated with AD and MCI pathology.

**Funding**: NSF‐2032709/Li; NIH‐1R21AG046637‐01A1/Kavcic; NIH‐1R01AG054484‐01A1/Kavcic; NIH‐P30AG072931/Paulson and NIH‐P30AG024824/Yung.

